# Influenza classification from short reads with VAPOR facilitates robust mapping pipelines and zoonotic strain detection for routine surveillance applications

**DOI:** 10.1093/bioinformatics/btz814

**Published:** 2019-11-06

**Authors:** Joel A Southgate, Matthew J Bull, Clare M Brown, Joanne Watkins, Sally Corden, Benjamin Southgate, Catherine Moore, Thomas R Connor

**Affiliations:** 1 Organisms and Environment Division, School of Biosciences, Cardiff University, Cardiff CF10 3AX, UK; 2 Public Health Wales, University Hospital of Wales, Cardiff CF14 4XW, UK; 3 MRC Centre for Regenerative Medicine, University of Edinburgh, Edinburgh EH16 4UU, UK

## Abstract

**Motivation:**

Influenza viruses represent a global public health burden due to annual epidemics and pandemic potential. Due to a rapidly evolving RNA genome, inter-species transmission, intra-host variation, and noise in short-read data, reads can be lost during mapping, and *de novo* assembly can be time consuming and result in misassembly. We assessed read loss during mapping and designed a graph-based classifier, VAPOR, for selecting mapping references, assembly validation and detection of strains of non-human origin.

**Results:**

Standard human reference viruses were insufficient for mapping diverse influenza samples in simulation. VAPOR retrieved references for 257 real whole-genome sequencing samples with a mean of >99.8% identity to assemblies, and increased the proportion of mapped reads by up to 13.3% compared to standard references. VAPOR has the potential to improve the robustness of bioinformatics pipelines for surveillance and could be adapted to other RNA viruses.

**Availability and implementation:**

VAPOR is available at https://github.com/connor-lab/vapor.

**Supplementary information:**

Supplementary data are available at *Bioinformatics* online.

## 1 Introduction

Influenza viruses are enveloped, single-stranded, segmented negative-sense RNA viruses of the family Orthomyxoviridae. Influenza A and B have eight genome segments, with major antigenic recognition sites within the two proteins haemagglutinin (HA) and neuraminidase. Accumulation of point mutations within the antigenic recognition sites of HA and neuraminidase can result in host immune evasion, thereby causing annual seasonal epidemics (Petrova and Russell, 2018; Taubenberger and Kash, 2010). Current estimates suggest that seasonal influenza A and B cause 4–5 million severe infections (Tafalla *et al.*, 2016) in humans with ∼291 000–645 000 (Iuliano *et al.*, 2018) deaths per year globally. Furthermore, influenza type A is a zoonotic virus infecting a wide range of avian and other non-human species (Sautto *et al.*, 2018). These viruses have the capability to reassort leading to the emergence of new strains (Bouvier and Palese, 2008), which can result in pandemics.

Whole-genome sequencing (WGS) has been used to study the influenza virus genome for over a decade and is emerging as an important tool in research and surveillance (Holmes *et al.*, 2005; McGinnis *et al.*, 2016; Meinel *et al.*, 2018; Rutvisuttinunt *et al.*, 2013). Protocols have been developed (Zhou *et al.*, 2009, 2014) that facilitate routine monitoring of isolates by public health organizations, as well as the study of transmission events (Houlihan *et al.*, 2018; Meinel *et al.*, 2018). Two important data sharing resources exist to this end; the NCBI Influenza Virus Resource (NIVR) (Bao *et al.*, 2008), and the Global Initiative on Sharing All Influenza Data (Shu and McCauley, 2017), wherein over a hundred thousand influenza genome segment sequences can be found at the time of writing. Sequencing can be performed directly from clinical swabs with single-reaction genomic reverse transcription polymerase chain reaction (RT-PCR) (Goldstein *et al.*, 2017; Zhou *et al.*, 2009). Furthermore, bioinformatics pipelines have begun to be developed for efficient processing of this data (Borges *et al.*, 2018; Wan *et al.*, 2015).

Despite the increasing application of next-generation sequencing to influenza, the pitfalls associated with current bioinformatics approaches have not been explored in depth. Influenza virus *de novo* assembly also poses additional challenges due to biological population complexity and additional error resulting from RT-PCR (Hunt *et al.*, 2015; Orton *et al.*, 2015). Firstly, we aim to provide evidence that current mapping approaches can, due to diversity of influenza genome sequences, routinely result in unmapped reads and potential data loss. This has been previously noted in study of human immunodeficiency virus (Wymant *et al.*, 2018). If reads are lost during mapping, coverage may appear to be poor, and downstream analyses, such as SNP analysis, estimates of viral intra-host diversity or even reconstructed sequences, may be biased. Furthermore, in the worst case, sequences of zoonotic origin may fail to be identified, resulting in a dataset that appears to be low coverage, missing segments or missing potential future pandemic reassortments. Whilst alternatives, such as read classification by mapping to a large database of influenza sequences (Yu *et al.*, 2014) and subsequent *de novo* assembly can help to resolve this issue, such pipelines are often complex, slow and require expertise that is not necessarily available in routine surveillance. Furthermore, even with recent assembly programs, misassembly can occur (Wymant *et al.*, 2018). We aim to show that this problem can be resolved by classification of isolates from reads prior to analysis by directly querying a De Bruijn graph (DBG) built directly from Illumina sequencing reads. Mapping reads directly to a DBG has been previously argued to be less biased than that of mapping to assembled contigs (Limasset *et al.*, 2016). Directly querying DBGs instead of assembled sequences has been previously addressed (Holley and Peterlongo, 2012; Limasset *et al.*, 2016; Liu *et al.*, 2016; Salmela and Rivals, 2014), although most previous work has focussed on mapping reads to a DBG, and not diverse RNA virus gene sequences. Instead of mapping reads to a DBG, we sought to develop a method for querying short influenza genome sequences against a short-read DBG in order to retrieve the most similar reference. We compare our tool, VAPOR, with both a slow BLAST-based (Altschul *et al.*, 1990) approach and fast *k*-mer-based MASH (Ondov *et al.*, 2016), and show superior or equivalent results in several use cases with reasonable runtimes.

## 2 Materials and methods

### 2.1 WGS datasets

Total RNA was extracted from patient samples using the NucliSens easyMAG instrument according to the manufacturer’s instructions. Following RNA extraction, a one-step RT-PCR (Quanta biosciences qScript XLT kit, following manufacturer’s instructions) was then undertaken to generate DNA for sequencing using the primers previously described for influenza A (Zhou *et al.*, 2009) and influenza B (Zhou *et al.*, 2014). Sequencing was performed using Illumina sequencing instruments. Libraries were prepared using NexteraXT, and samples were then multiplexed for sequencing. Samples were run on a MiSeq (2 ×250 bp V2 kit—44 samples) and NextSeq (2 ×150 bp Medium Output kit—213 samples). In total, 257 samples were utilized. Short-read data can be found at s3.climb.ac.uk/vapor-benchmark-data/vapor_benchmarking_realdata_reads_filtered_18_03_18.tar, or hosted at the European Nucleotide Archive under project accession PRJEB33950.

For publicly available data, any reads that were classified as human by Kraken2 (Wood and Salzberg, 2014), or those that mapped to the hg38 human genome with Minimap2 (Li, 2018), were removed.

These WGS datasets were then processed by extraction of influenza reads by mapping with Minimap2 (Li, 2018) to eight curated influenza segment reference.fasta files (19 594 sequences in total), one at a time. These reference files were prepared by downloading all available influenza segment sequences from the NIVR (https://www.ncbi.nlm.nih.gov/genomes/FLU/) and clustering to 99.5% identity with cd-hit-est (Li and Godzik, 2006). Extracted reads were assembled with IVA (Hunt *et al.*, 2015). For all 257 fastq file pairs used, a near-full-length (>90%) contig could be assembled for at least one major segment protein. Samples for which a contig could not be assembled were not used. In total, 1495 segment contigs were included.

### 2.2 Mapping assessment

Four mapping programmes were assessed in this analysis: Minimap2 (Li, 2018), BWA-MEM (Li and Durbin, 2009), NGM (Sedlazeck *et al.*, 2013) and Hisat2 (Kim *et al.*, 2015). These tools were used to represent a range of algorithms and intended use cases, in order to assess whether robustness to influenza data is a common problem, or whether it can be addressed by choice of tool. Default settings were used for all tools. Each experiment can be reproduced using the code and instructions found at github.com/connor-lab/vapor_mapping_benchmarking. Four mapping simulations were performed in total.

For assessment of the sufficiency of single reference strains for mapping diverse samples, two simulations were performed. For assessment of robustness to species origin, read sets were simulated with ArtificialFastqGenerator (Frampton and Houlston, 2012) from 552 avian, 16 679 human and 4054 swine H1N1 HA coding sequences from the NIVR (Bao *et al.*, 2008). An additional 0.05% *in silico* substitution was introduced into simulated reads to account for RT-PCR technical errors and biological intra-host variation. This rate was chosen to be in accordance with experimental observations made by Orton *et al.* (2015). Reads were then mapped to the A/California/07/2009 (H1N1) HA reference sequence. For assessment of robustness to divergence, technical and biological noise, reads were simulated from A/Perth/16/09 (H3N2) HA, with additional *in silico* mutation with per-base rates between 2 and 16%, performed uniformly across the chosen reference sequence; reads were simulated as above, then mapped back to A/Perth/16/09 (H3N2). This was performed 1000 times for each mutation rate. A/California/07/2009 (H1N1) and A/Perth/16/2009 (H3N2) were used as references since they are common clade representatives. Samtools (Li *et al.*, 2009) was used to retrieve successfully mapped reads, which were then counted.

For comparison of mapping with and without VAPOR classification, and potential zoonotic virus detection, two simulations were performed using 33 133 unique approximately full-length influenza A HA coding sequences of any lineage or species, downloaded from the NIVR. In the first case, 5000 pairs of sequences were chosen randomly; the first of the pair was used for read simulation as above, and the second as a mapping reference. In the second simulation, a single sequence was randomly chosen for read simulation, and the reference was chosen by VAPOR version 1.0.1. This process is shown in Supplementary Figure S1. As before, successfully mapped reads were extracted with samtools, then counted.

To assess the potential benefit of classification with VAPOR on real data, 206 of 257 fastq file pairs were subjected to mapping with Minimap2 with default settings for short reads (-x sr), both with and without VAPOR classification. A total of 51 out of 257 samples with <1000 HA reads were excluded to avoid very low coverage samples skewing calculation of mean percentage gain. In the first case, reads were mapped to a set of 4 HA references from different subtypes: A/Perth/16/2009 (H3N2), A/California/07/2009 (H1N1), B/Florida/4/2006 (Yamagata) and B/Brisbane/60/2008 (Victoria). In the second case, VAPOR was used to choose a single reference from 53 758 influenza A and B HA references. The number of reads mapping and the number passing VAPOR pre-filtering was recorded in each case.

### 2.3 Algorithm overview

#### 2.3.1 Definitions

Let $R=\{r_{1},r_{2},\ldots ,r_{\left|R\right|}\}$ and $S=\{s_{1},s_{2},\ldots ,s_{\left|S\right|}\}$ be indexed multisets of strings (sequencing reads and references, respectively), over a common alphabet Σ={A,T,C,G}, where |*R*| denotes the cardinality of set *R*. Let W=(N,E,W) be a weighted DBG built from reads *R*, where *N*, *E* and *W* are sets of nodes (*k*-mers), edges (*k*−1-mers) and node weights (sequencing depth for some *k*-mer), for some k≥2 (by default *k *=* *21). We assume a model read generation process reflective of RNA virus sequencing: let the multiset $X=\{x_{1},x_{2},\ldots ,x_{\left|X\right|}\}$, be a population of virus sequences (quasispecies) for some gene, for which we suppose there is some major variant x* with the greatest multiplicity. Let reads *R* be generated from this population, with varying coverage across the gene (possibly by several orders of magnitude), and additional errors (due to RT-PCR and sequencing). We attempt, using heuristics, to find a reference that is similar to x*.

#### 2.3.2 Mapping and scoring

**Fig. 1.**
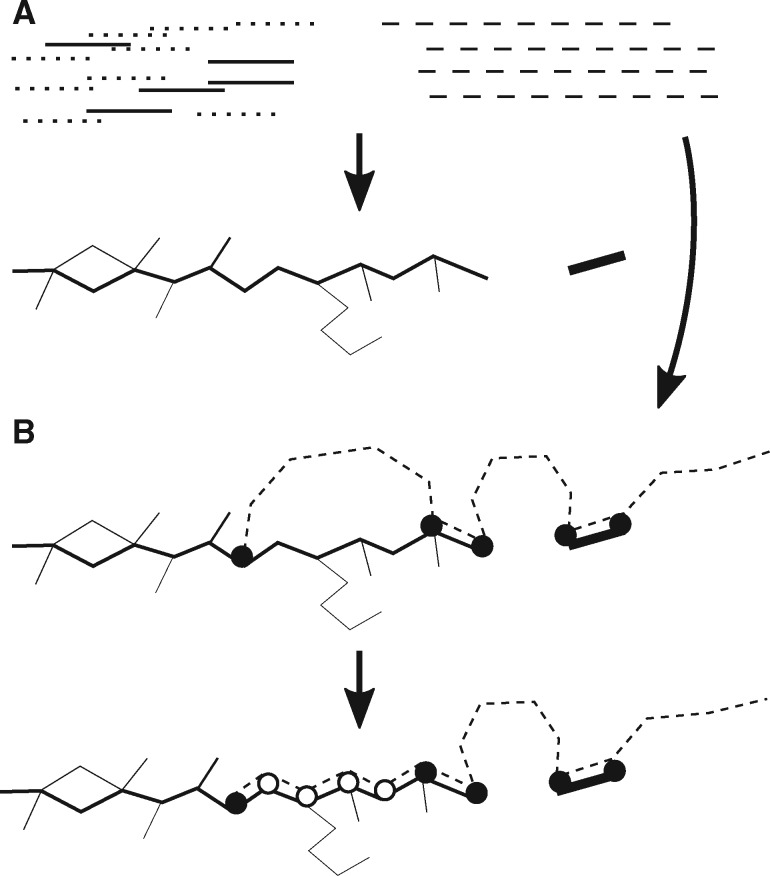
Simplified VAPOR algorithm. Firstly, pre-processing and graph construction is performed (**A**), where target reads *R* (solid black lines) are filtered from non-target (e.g. bacterial) reads (dotted lines) using a fast *k*-mer comparison to references *S*. This is followed by wDBG construction. Then, mapping and scoring is performed simultaneously (**B**), where each reference sequence *s* (dashed line) is mapped to the wDBG, W, built from these reads. This is done in two main steps: exact *k*-mer matching (black circles) and extension (white circles) by heuristic graph traversal

VAPOR maps each reference *s* against W, such that the *i*th *k*-mer of *s*, denoted *s*[*i*, *i *+* k*] is either mapped to some node n∈N, or mapped to a gap. We note that *s*[*i*, *i *+* k*] does not have to equal *n*. Let *s′* be the string representation of the path mapped to by *s*. Figure 1 demonstrates the concept of this mapping.

We next formulate a scoring function $f_{W}(s,s\prime )$. We chose to favour sequences for which there is high weight in W; due to the high degree of variation in RNA virus datasets, and large number of closely related reference sequences, many *k*-mers may be present in the W at low frequency, such that there may be several reference sequences which correspond exactly to a path in W. Conversely, since sequencing depth in these datasets may be highly skewed, we seek to also reward matches which cover a greater proportion of the reference, rather than those that have high depth for a short subsequence, then poor matches elsewhere. In order to capture this trade-off, we define:

(1) $$f_{W}(s\prime ,s)=\psi (s\prime )\cdot \sum_{i=1}^{\left|s\right|}M_{i}\delta (s\prime_{i},s_{i})$$

where ψ(s′) is the fraction of non-gap bases of s′, |s| is the length of string *s* and *M_i_* is the maximum sequencing depth of *k*-mers that overlap with the *i*th base of *s^′^*. That is, for the sequence of node weights *w_i_* with corresponding *k*-mer nodes *n_i_* of $s\prime ,\;M_{i}=\max\{w_{\bar{i}},w_{\bar{i}+1},\ldots ,w_{i}\}$, where $\bar{i}=\max(0,i-k+1)$ and $\delta (s\prime_{i},s_{i})=1$ if $s\prime_{i}=s_{i}$, and 0 otherwise. Since any reference can be mapped onto the graph in many ways, we attempt to heuristically find high scoring placements.

#### 2.3.3 Pre-processing

VAPOR first filters reads to remove non-target sequences (e.g. bacterial) and decide orientation of reads. As input, VAPOR takes a fasta file of full or approximately full-length reference segment sequences, and a.fastq (or.fastq.gz) file of WGS reads. Firstly, VAPOR builds a set of *k*-mers *U* from all reference sequences. Next, the *i*th read is decomposed into a set of non-overlapping subsequences of length *k* (words), *A_i_*, and if $|A_{i}\cap U|/|A_{i}|\leq t$ (the proportion of read words also present in the references), where |*A*| gives the number of elements of the set *A*, for some specified parameter *t*, the read is discarded. This is repeated for the reverse complement; if both are kept, the highest score decides orientation. Furthermore, in order to try to eliminate erroneous *k*-mers, any node $n_{j}\in N$ with corresponding weight $w_{j}\in W$ less than a coverage parameter *c* is discarded.

#### 2.3.4 Core algorithm

**Fig. 2.**
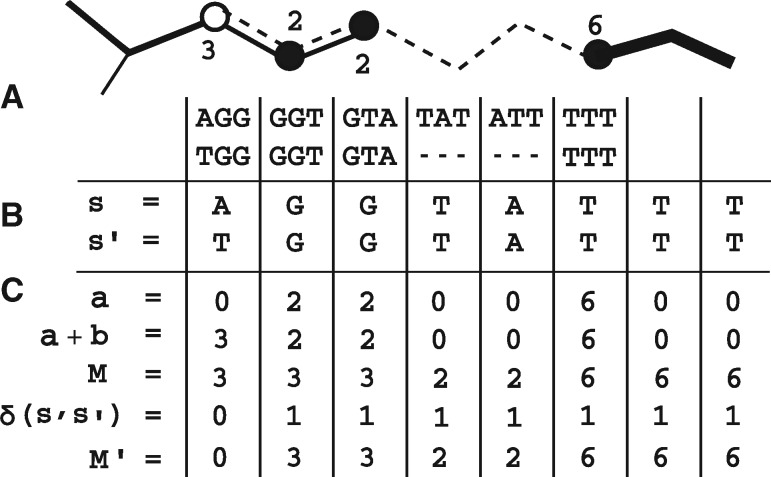
Scoring procedure for an example graph mapping. An example mapping of a reference *s* (dashed line) to a graph W (solid lines), with exact matches (black circles) and inexact matches (white circles), is shown (**A**), with string representation of the path, *s^′^* (**B**). Firstly, a weight array *a* is retrieved for which *a_i_* gives the weight of the *i*th reference *k*-mer in the wDBG, where gaps are given a weight of zero. These exact matches are then extended with bridges *b* to inexact matches. Next, per-base weights *M* are calculated such that each base is given the greatest weight of any *k*-mer that includes it, which also functions to assign weights to terminal characters of a string (or substring before a gap) that do not have *k*-mers (such as ‘TA’ at the fourth position). Finally, the array *M^′^* is computed as $M_{i}^{\prime }=\delta_{i}\cdot M_{i}$, where $\delta_{i}=1$ if $s\prime_{i}=s_{i}$, and zero otherwise. For our classifier, we chose to multiply the sum of this array by the fraction of non-gap (non-zero) positions, in order to penalize high weight, high gap mappings

Firstly, W is built from the filtered reads. Then for each input reference sequence, *s*, the core algorithm of VAPOR makes use of a heuristic seed-and-extend procedure to find a high scoring mapping of *s* onto W. Each reference sequence, *s*, with length |*s*|, is decomposed into a sequence of *k*-mers. Querying proceeds in four phases, where the query is walked along the wDBG: *k*-mer seeding, trimming, bridging and scoring. We seek to simultaneously perform the mapping and compute the array $M\prime =(M_{1}\delta (s_{1},s_{1}^{\prime }),M_{2}\delta (s_{2},s_{2}^{\prime }),\ldots ,M_{\left|s\right|}\delta (s_{\left|s\right|},s_{\left|s\right|}^{\prime }))$ as in (1). Firstly, an array *a* is initialized from exact *k*-mer matches, where *a_m_* is the weight of the *m*th *k*-mer of the reference, and any not in *N* are set to zero. For speed considerations, only a subset of seed arrays is extended: those with a fraction of non-zero elements greater than a user-defined parameter –min_kmer_cov (default: 0.1), and in a top user-defined percentile –top_seed_frac (default: 0.2). In order to reduce the number of suboptimal exact matches, seeds are trimmed. Each seed (sequence of *k*-mer matches) in the array *a*, is trimmed back (set to zero) at both ends until a suboptimal branch points in the graph within *ρ* positions of the end of the seed is found. This procedure is used to heuristically prevent suboptimal seeds to low coverage regions of the wDBG, possibly generated by error or low frequency variants. Next, bridging is performed. For the *i*th gap (run of zeros) in *a* of length *l*, a bridge *b_i_* is formed by walking *l* locally optimal (where there is a branch, the edge with the highest weight) edges in the wDBG from the last matching *k*-mer. As such, bridging attempts to extend a mapping with only exact matches to one with inexact matches. Next, the array *M* is computed by (i) inserting bridge *k*-mer weights and (ii) re-calculating the weight at each position *j* as *M_j_* (as defined in mapping and scoring). Finally, each bridge, *b_i_*, a string, is then compared to the *i*th gap string, the original substring in the reference sequence corresponding to the gap, in order to compute $\delta (s\prime_{j},s_{j})$ as in (1). For any *s_j_* in an exact match, $\delta (s\prime_{j},s_{j})=1$ by definition. Figure 2 shows the steps involved in computing the array *M^′^* for an example graph mapping.

VAPOR is implemented in Python3, with source code available at github.com/connor-lab/vapor.

### 2.4 Classification benchmarking

VAPOR was compared to MASH (Ondov *et al.*, 2016) and BLAST (Altschul *et al.*, 1990) read classification by simulation. BLAST consensus classification was performed by BLASTing each read, taking the best scoring references by *e*-value then bit-score, summing the number of times each result occurs in all reads and returning the most frequent. Reads were simulated as follows: a reference, *s*_0_, was chosen from 46 724 unique approximately full-length influenza A HA sequences from the NIVR, and mutated uniformly with a given probability (0.01, 0.02, 0.03) to generate a mutated sequence *s_m_*; reads were simulated with ArtificialFastqGenerator as before, with a higher uniform error rate of 1%, in order to provide a challenging classification task representative of difficult datasets. To provide an additional challenge, we simulated an intra-host population with four minor sequences, mixed in the ratio of 100:5:1:1:1, with each minor sequence additionally mutated by 1% relative to the major sequence.

This process was performed 500 times for each category. Performance was assessed as follows: the Levenshtein distance of the mutated sequence *s_m_* was taken with respect to the original sequence *s_o_* as a baseline, denoted by $L(s_{m},s_{o})$; the reads were classified by each tool with all 32 804 references as a database, and the best hit *s_c_* returned by each were compared to the mutated sequence to obtain *L*(*s_m_*, *s_c_*). Global alignment was performed with the pairwise2 module of Biopython (Cock *et al.*, 2009) (with cost parameters 0, −1, −1, −1, corresponding to Levenshtein distance). We defined the additional Levenshtein distance, $L_{A}=L(s_{m},s_{c})-L(s_{m},s_{o})$. This distance was chosen because, for mutated sequences, it captures the additional error in classification beyond that caused by uniform mutation to the original reference. We note that *L*(*s_o_*, *s_m_*) may occasionally be suboptimal, i.e. there may exist *s_o_* such that $L(s_{o},s_{m})<L(s_{o},s_{m})$ where *in silico* mutations introduced resulted in a sequence more similar to some other sequence in the database than the original.

For real datasets, 257 raw fastq file pairs that produced full-length contigs for at least one segment were chosen from the sequencing runs described above. The assembled contigs were annotated with BLAST (sorting by *e*-value, bit-score and length), and raw reads classified by VAPOR. The percentage identity (PID) of VAPOR classifications to each contig was recorded.

### 2.5 Detection of reassortments and zoonotic strains

For assessment of reassortment classification, two simulations were performed. Firstly, 9659 avian, 18 308 human and 2893 swine complete influenza genome sets were downloaded from the NIVR and 250 influenza human genome sets were randomly selected. Another 250 were randomly selected with a single segment swapped with a randomly chosen avian or swine influenza segment. For each, 1000 reads from each segment were simulated uniformly with an error rate of 0.5%. Each set of reads was classified with VAPOR. For the reference strains chosen by VAPOR for each segment, respective HA sequences were compared by global alignment, and PID taken. If the maximum pairwise distance between chosen strain HA sequences exceeded a given threshold *v*, a classification of true was returned. Receiver operating characteristic curves were generated by varying the parameter *v*. For assessment of intra-subtype reassortment classification, the same experiment was performed with randomly chosen H3N2 genomes.

### 2.6 Computational resources and performance benchmarks

In all cases, experiments were performed natively on a 96 core, 1.4 TB memory CentOS version 7.4.1708 virtual machine hosted by Cloud Infrastructure for Microbial Bioinformatics (CLIMB) (Connor *et al.*, 2016), with GNU parallel (Tange, 2011) where required. Basic time and space benchmarks comparing BLAST and VAPOR were performed with GNU-time (wall-clock time, maximum resident set size). The largest 11 influenza A samples with at least 1% (between 1 and 16%) of reads identifiable as influenza (samples 36, 51, 93, 95, 100, 101, 105, 113, 114, 131, 155) were randomly sub-sampled to between 200 000 and 2 000 000 reads (total for a pair of fastq files). BLAST and VAPOR were used to query a database of 47 073 influenza A HA gene sequences. For BLAST, reads were first converted to fasta files, which was not included in the benchmark time. For BLAST, tabulated output was specified (-outfmt 6), and output redirected to/dev/null in order to reduce I/O time (assuming a tool using BLAST for classification would not parse output files). VAPOR was also benchmarked on a laptop with an Intel^®^ Core^TM^ i7-6600U CPU @ 2.60 GHz with 8 GB of memory, using the same samples without sub-sampling (mean read total 6 600 000).

## 3 Results

### 3.1 Benchmarking single-reference mapping

**Fig. 3.**
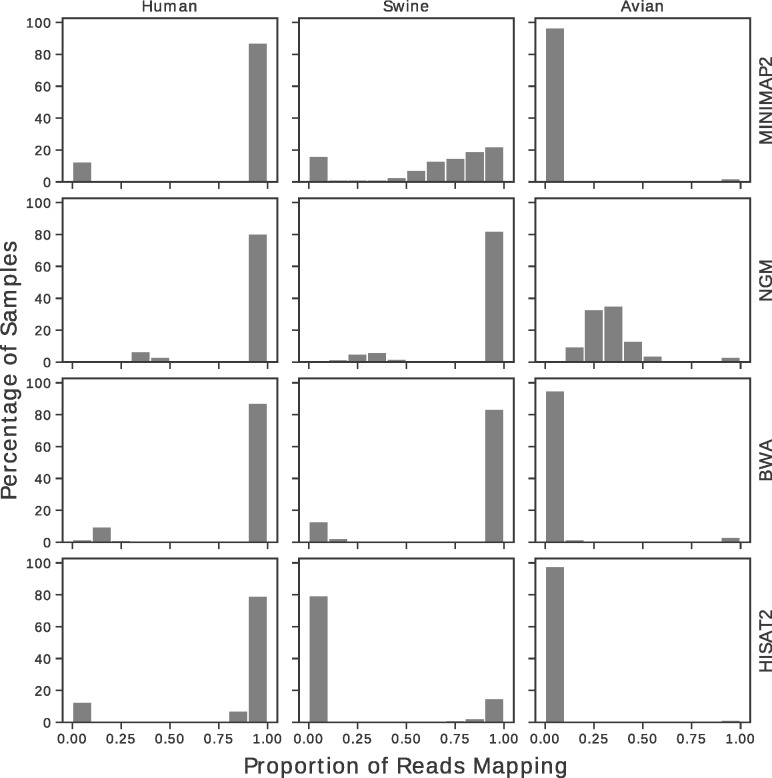
Histogram showing proportion of mapped reads, by software and dataset. Reads were simulated for each dataset retrieved from the NIVR: 16 679 Human H1N1 HA (left column); 552 avian H1N1 HA (middle column); 4054 Swine H1N1 HA (right column). All sequences were mapped to California/07/2009. For human viruses, most simulated datasets mapped successfully, although even for this dataset, around 10% of samples had some proportion of unmapped reads. However, for avian and swine sequences, mapping quality was poor, and often failed entirely. Even for the best performing software, NGM, avian sequences mapped poorly

A range of mapping programmes (Minimap2, BWA-MEM, Hisat2 and NGM) were compared to assess possible data loss when single references are chosen for mapping of short reads from influenza virus WGS datasets. For the first experiment, simulated reads from 16 679 human, 552 avian and 4054 swine H1N1 HA sequences retrieved from the NIVR were mapped to the reference strain A/California/07/2009 (H1N1). Reads were simulated with an additional 0.05% error on top of simulated sequencing error to account for the combined effect of intra-host population variation and RT-PCR error. This error rate was found to be conservative when compared to the raw error rate in our datasets, as shown by Supplementary Figure S3, which was frequently higher than 2%. The proportion of successfully mapped reads for each tool and host species is given in Figure 3. In this case, using a single reference strain with any of the programmes resulted in unmapped reads. NGM resulted in the lowest average percentage of unmapped reads. When utilizing a database of all H1N1 sequences from human hosts, Minimap2, NGM, BWA-MEM and Hisat2 had mean mapping percentages of 87.2, 92.2, 89.1 and 84.9% respectively; as such, even for these influenza sequences, data loss was not uncommon, possibly due to samples in the database representing human infection from zoonotic strains. However, for avian and swine samples, read recovery was poor. For NGM, only 34.1% of avian reads mapped successfully on average. Swine sequences were mapped with intermediate success. This provides evidence that, should zoonotic strains be sequenced in routine surveillance, they may fail to map entirely, and go undetected.

Secondly, in order to assess how read recovery varies with sequence divergence, reads were simulated by taking the coding sequence of A/Perth/16/09 HA and subjecting it to *in silico* uniform mutation at specified rate, with additional read error of 0.05% as before (Supplementary Fig. S4). For all mapping programmes, at ∼10% mutation, read recovery begins to regularly diminish, which is insufficient for robust mapping of influenza strains from different species. Furthermore, for several of the programmes tested, mapping quality was suboptimal beyond 1–3% mutation.

### 3.2 Simulation classification performance

**Fig. 4.**
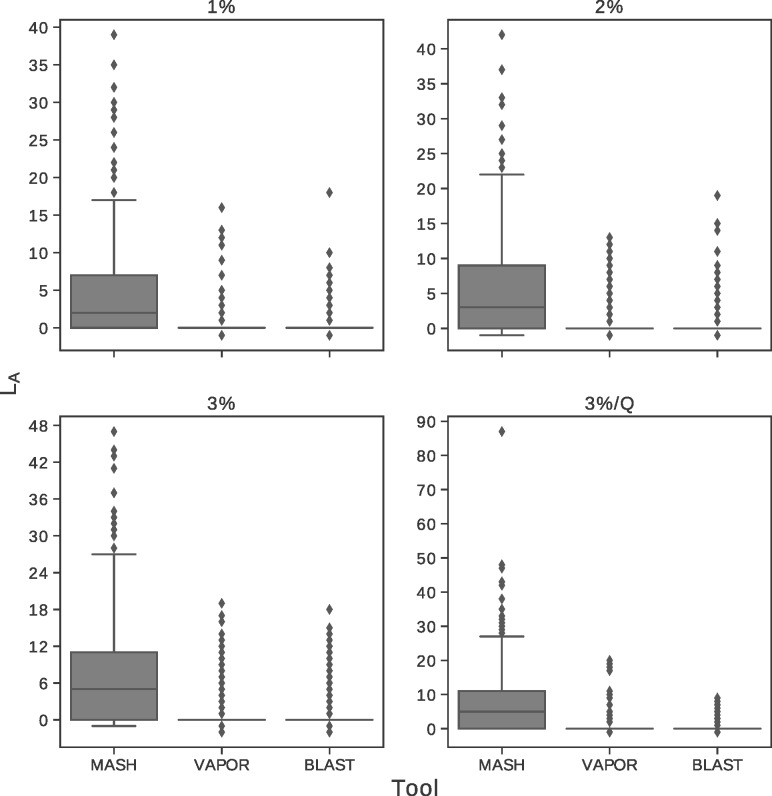
Box plots showing additional Levenshtein distance of input sequence to output reference chosen by VAPOR, MASH and BLAST consensus classification. Reads with 1% error rate were generated from randomly selected references mutated *in silico* by 1, 2, 3 and 3% with additional biological intra-host variant noise simulation 3%/Q, and repeated 500 times for each category. *L*_A_ is defined as Levenshtein distance of a classified sequence *s*_c_ to original mutated sequence *s*_m_, minus the distance of the original mutated sequence *s*_m_ to the original non-mutated reference sequence *s*_o_. Outliers are indicated as diamonds. Performance of VAPOR was generally equivalent to that of BLAST. For both of these tools, classification most often resulted in none, or a few extra incorrect bases

In order to assess the performance of classification from simulated reads, our tool, VAPOR, was compared to MASH and consensus BLAST classification. Reads were simulated from randomly selected H1N1 HA sequences mutated with a given uniform per-base probability, with additional read error of 1% to provide challenging datasets. A fourth category included simple simulated intra-host populations (denoted as 3%/Q). Figure 4 shows the additional Levenshtein distance, *L*_A_, for each tool. Mean coverage for simulated reads was 77.76 for single-sequence simulations, and 96.03 for simulated intra-host populations. The average additional distance of retrieved sequences for MASH were 4.69, 5.24, 6.83 and 7.28, showing some sensitivity to additional simulated variant noise; for all cases mean additional distance for BLAST and VAPOR were below 0.74 and 0.88, respectively. For MASH, the 75, 95 and 99% percentiles for retrievals for the 3% threshold were 11.00, 24.00 and 37.04. However, for BLAST and VAPOR, these percentiles were under 12 and 14, respectively, for all cases. These results show that references chosen by BLAST and VAPOR were often near-optimal or optimal, despite a large amount of noise, and that the performance difference between these approaches was small. These results show that the algorithm used by VAPOR facilitates accurate classification of influenza strains directly from reads, comparable in accuracy to BLAST for WGS read sets, which is generally not computationally tractable for datasets with millions of reads (shown in Supplementary Fig. S7).

### 3.3 Real data classification performance

**Fig. 5.**
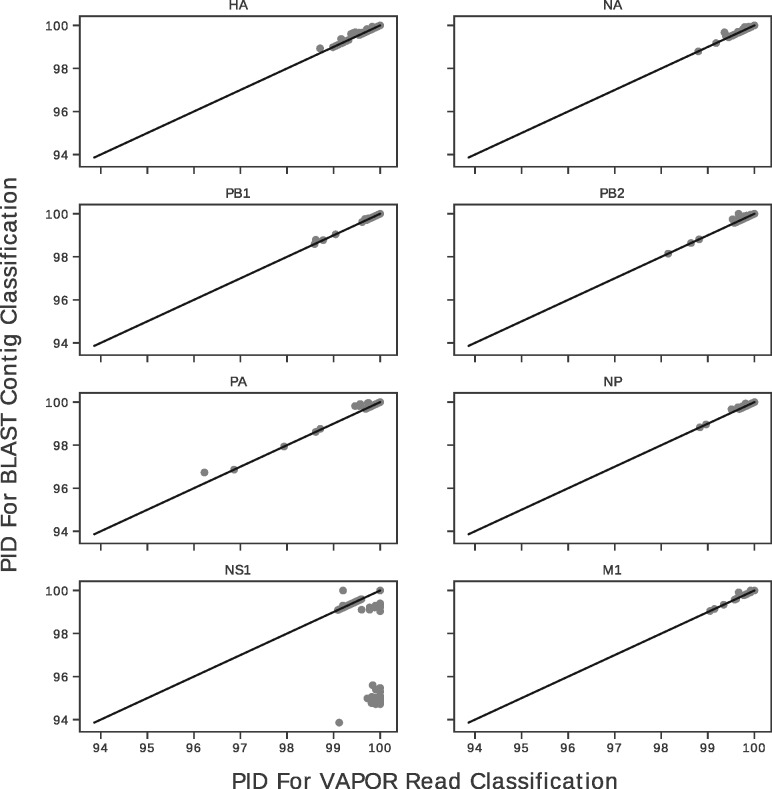
Scatterplots showing PIDs of VAPOR read classifications versus BLAST contig classifications with respect to assembled contigs for all eight major segment coding sequences. Black lines indicate *x *=* y*. Points that fall below this line were classified better from reads with VAPOR. Points above the line were classified better with BLAST from contigs. VAPOR is capable in general of performing classification of reads to within 1% of the correct sequence. The mean PID of VAPOR classifications for all segments was 99.82%. For datapoints under 98% PID, BLAST was generally also not able of providing a better classification given the reference database

In order to validate the performance of VAPOR directly on real datasets, we took raw reads from 257 samples corresponding to 1495 segment contigs previously processed and assembled with IVA, with a single full-length contig each previously annotated by BLAST. In each case, corresponding reads were classified by VAPOR. The chosen reference was compared by global alignment to the assembled full-length contigs. Figure 5 gives a scatter plot showing the PID of references retrieved by VAPOR to the assembled contig versus the PID of references selected by BLAST classifications of contigs. Comparison to BLAST classification of contigs was used to provide a baseline near-optimal classification. The mean PID between contig and VAPOR classification was 99.82%. In the case of NS1, VAPOR outperformed BLAST annotation of assembled contigs, with a mean of 99.48 versus 98.74%. On closer inspection, this was a result of the method used to sort BLAST results combined with the presence of a sequence with an additional 150–200 bp of the 3′ untranslated region (UTR). Removal of this sequence and sorting by PID resulted in a mean of 99.45 and 99.93%, respectively. These results show that in most cases tested, VAPOR was able to accurately identify a sample from reads with comparable performance to BLAST annotation of assembled contigs. We note that, for some contigs, neither BLAST nor VAPOR could achieve classifications with a PID >97%. Manual examination of these samples showed large deletions, with at least one a likely misassembly (deletion including start codon, inclusion of 5′ UTR).

### 3.4 Mapping with pre-classification

**Fig. 6.**
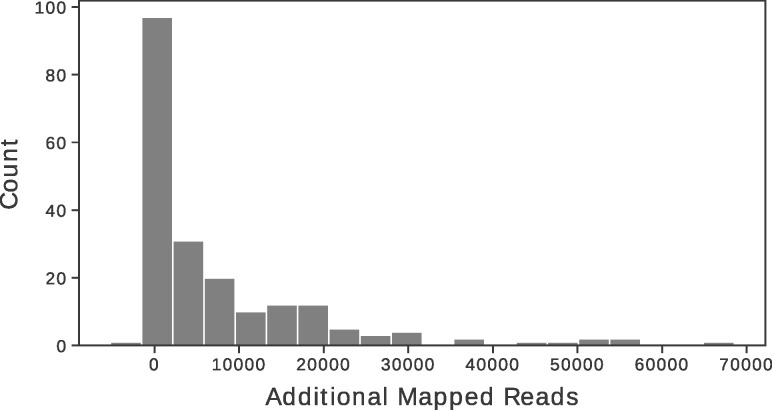
Additional number of reads mapped by Minimap2 with VAPOR pre-classification for 257 real.fastq file pairs. Pre-classification with VAPOR on average resulted in 7816.03 more mapped reads. Several samples gained more than 50 000 reads by choosing a suitable reference. For one sample, representing a possible coinfection, 5221 fewer reads mapped when using a single reference chosen by VAPOR

Raw mapping performance was also assessed on real data by mapping datasets with Minimap2 with and without pre-classification. Figure 6 shows the number of additional reads mapped when pre-classification was performed. In all but one case, this resulted in a greater number of mapped reads, with a mean of 7816.03, corresponding to a mean percentage gain of 6.85%, including a case with over 68 000 additional reads. The maximum percentage increase was 13.32%. An outlier did occur where the number of mapped reads decreased. In this case, VAPOR identified several thousand more reads as influenza than were mapped. On further inspection, for this sample, reads mapped to both A/Perth/16/09 (H3N2) and A/California/07/09 (H1N1), indicating that the sample represented influenza from two different subtypes. As such, this sample represented a true biological coinfection or a contamination and could not be mapped to a single reference.

For simulations with randomly selected pairs from any species, mean recovery rates were <20% without pre-classification (Supplementary Fig. S5). However, with pre-classification using VAPOR, the mean was over 99.72% for all tools, demonstrating that mapping with pre-classification is robust to sequences of non-human origin.

### 3.5 Detection of reassorted strains directly from reads

In order to assess the application of read pre-classification to reassortment detection directly from reads, 250 simulated reassortment events with zoonotic strains were mixed with 250 complete genome sets, reads simulated, then classified by VAPOR. A simple reassortment classifier was used on the output of VAPOR, which compared the minimum pairwise PID of the HA sequences of the eight strains assigned by VAPOR to each segment; if this PID was below a given parameter *v*, a reassortment was called. A receiver operating characteristic curve is shown in Supplementary Figure S6, illustrating the performance of this classification strategy. Simulated zoonotic reassortments were detected with 97.2% true positive rate and 0.08% false positive rate for a *v* of 91.35%. This is expected because, as previously shown, VAPOR generally was able to classify strains to within a few base-pairs; randomly chosen zoonotic strains generally had PIDs of <90% to human strains, depending on origin. We note that, given the database used, some avian strains may have been isolated from humans, and labelled as human; as such, perfect classification with this dataset may be impossible. In order to provide a more difficult reassortment detection task, the same experiment was performed between human H3N2 sequences. We found at a PID threshold of 96.3%, a true positive rate of 76.8% could be achieved at a false positive rate of 10.8%. This result was expected given that sequences from different H3N2 strains generally have a PID within a few percent. In total, these results provide evidence that reassortments with zoonotic strains can be detected directly from reads with reasonable accuracy, but that intra-lineage reassortments may be more difficult.

### 3.6 Run-time and computational requirements

For benchmarking performed on the CLIMB VM, VAPOR performed all benchmarks within 11 min. As expected, using BLAST to query all reads was too slow for general application to these datasets, as shown in Supplementary Figure S7. For the 2 000 000 read samples, BLAST took over 20 h in the worst case, excluding both pre- and post-processing of results. For the laptop computer, VAPOR classified samples with a mean of 6 600 000 total reads in a mean time of 3.73 min and mean peak memory consumption of 2.78 Gb.

## 4 Discussion

### 4.1 Mapping approaches and improvement with VAPOR

We provide evidence that, in the best case, approaches for influenza virus analysis that use mapping to a single reference may result in data loss due to biological variation and noise. As shown in Supplementary Figures S2 and S3, influenza strains continually accumulate substitutions relative to a single reference (∼5 substitutions per year for H3N2) and reads may have a high error rate (>2%). Often, mapping to a single reference may be most unreliable for important samples, such as zoonotic transmission events. In the worst cases, mapping may fail completely, when usable data are present, requiring time and expertise to resolve with more complex methods. Our approach largely avoids these pitfalls altogether, allowing simpler pipelines and retaining the advantages of using a mapping-based approach for analysis. We chose Minimap2, BWA-MEM, NGM and Hisat2 in order to represent a range of mapping softwares. BWA has found use in general for influenza read mapping (Borges *et al.*, 2018; Imai *et al.*, 2018; Jonges *et al.*, 2014; Leonard *et al.*, 2016; Rutvisuttinunt *et al.*, 2013; Wu *et al.*, 2014; Yu *et al.*, 2014). In some cases, references were chosen by mapping-based approaches for selection (Yu *et al.*, 2014). Of these softwares, only NGM was developed with specific robustness to variation. Furthermore, the experiments reported were not intended as complete evaluations of the programmes, since such an evaluation must also include mapping quality. Our data, however, does demonstrate that pre-classification with raw reads provides a broad strategy to improve robustness of pipelines and achieve faster results. For the chosen references, A/Perth/16/2009 (H3N2) and California/07/2009 (H1N1) were chosen as vaccine strains recommended by the WHO multiple times, and have also been used previously as references (Rutvisuttinunt *et al.*, 2013; Simon *et al.*, 2019). In other cases, different single references have been used (Meinel *et al.*, 2018). We do not believe that using different individual strains would affect the trends demonstrated.

We note that alternative approaches exist, including mapping to a large sequence database, but this does not easily allow for visualization of an alignment to a single reference, and subsequent analysis such as characterization of point mutations. We note that in principle, pre-classification with any software could work reasonably well. MASH performed well in simulations. However; using an optimal reference is ideal, since for later advanced applications, such as transmission events, or study of intra-host variation, the closest possible reference may be necessary. Furthermore, VAPOR permits simultaneous filtering out of any non-human or bacterial reads with optimal reference selection. Whilst BLAST performed well for individual read classification, it is often too slow for general application. Furthermore, assembly of virus genomes can be slow, often taking several days for a single sample when contaminant reads, such as human DNA, are present. Finally, misassembly can occur (Wymant *et al.*, 2018), which potentially occurred in several of our samples.

In all but one of our real-data cases examined, pre-classification with VAPOR resulted in a greater number of mapped reads than mapping to four reference strains from A/H3N2, A/H1N1, B/Victoria and B/Yamagata. However, for a single sample, which contained influenza sequences from two clades, the number of mapped reads was reduced. Although VAPOR can report the number of influenza sequences detected in total, future study should be utilized to develop methods of coinfection detection. In these relatively rare cases, a single reference is not sufficient for mapping.

### 4.2 VAPOR algorithm and performance

We have presented a novel approach to virus classification from short-reads data using DBGs. In future study, as public sequence data accumulates, our algorithm may show promise in WGS approaches for other RNA viruses with small genomes, such as measles virus, human immunodeficiency virus or Ebola virus. Several default parameters were explored during development, but not exhaustively. A *k*-mer size of 21 was utilized, as this was also able to perform read pre-filtering from contaminating sequences, without addition of a separate parameter. Similarly, parameters controlling the minimum fraction of required *k*-mers for seed extension, as well as the top percentile of seeds chosen for extension could be adjusted, possibly to improve speed. However, in the read sets examined, the default parameters were generally sufficient to ensure matches were found and did not appear to exclude potentially optimal matches. However, for novel strains that differ greatly from all strains previously observed, more sensitive parameterizations may be required.

### 4.3 Real-data classification

As shown in Figure 4, we note that the BLAST contig classification strategy we used performed poorly on NS1. This was due to sorting by *e*-value, bit-score and length over PID, combined with the presence of some NS1 sequences in the database which were longer than the required coding region. Since in general usage we do not wish to exclude sequences with longer 3′ UTRs, we opted to include this result to illustrate a potential pitfall that can occur with automated BLAST classification, as well as the trade-off between length and PID. Although sorting by PID may alleviate this problem, it may also yield shorter, incomplete alignments. For some samples, neither BLAST nor VAPOR could retrieve a sequence closer than 96% to the assembled contig. For some samples, this was due to large deletions present in the assembled contig. Although some of these deletions may be present in the true biological sequences, for at least one, this was due to suspected misassembly. These assemblies were also included to draw attention to potential problems that may be encountered during analysis.

## 5 Conclusion

Here we demonstrate that influenza sequence pre-classification with VAPOR minimizes data loss, reduces pipeline complexity, and allows for classification of zoonotic strains and reassortments directly from reads. We believe that the simplicity of our approach has potential to alleviate several difficulties associated with current bioinformatics pipelines and could reduce workloads in public health surveillance. Lastly, whilst we have tested VAPOR extensively for use with influenza, we believe our approach may be more broadly applicable to other sequence data, particularly small RNA and DNA viruses.

## Supplementary Material

btz814_Supplementary_Data

## References

[btz814-B1] Altschul S.F. et al (1990) Basic local alignment search tool. J. Mol. Biol., 215, 403–410. 2231712 10.1016/S0022-2836(05)80360-2

[btz814-B2] Bao Y. et al (2008) The influenza virus resource at the National Center for Biotechnology Information. J. Virol., 82, 596–601. 17942553 10.1128/JVI.02005-07 PMC2224563

[btz814-B3] Borges V. et al (2018) INSaFLU: an automated open web-based bioinformatics suite “from-reads” for influenza whole-genome-sequencing-based surveillance. Genome Med., 10, 46. 29954441 10.1186/s13073-018-0555-0 PMC6027769

[btz814-B4] Bouvier N.M., Palese P. (2008) The biology of influenza viruses. Vaccine, 26, D49–D53. 19230160 10.1016/j.vaccine.2008.07.039 PMC3074182

[btz814-B5] Cock P.J. et al (2009) Biopython: freely available Python tools for computational molecular biology and bioinformatics. Bioinformatics, 25, 1422–1423. 19304878 10.1093/bioinformatics/btp163 PMC2682512

[btz814-B6] Connor T.R. et al (2016) CLIMB (the cloud infrastructure for microbial bioinformatics): an online resource for the medical microbiology community. Microb. Genom., 2, e000086. 28785418 10.1099/mgen.0.000086 PMC5537631

[btz814-B7] Frampton M., Houlston R. (2012) Generation of artificial FASTQ files to evaluate the performance of next-generation sequencing pipelines. PLoS One, 7, e49110. 23152858 10.1371/journal.pone.0049110 PMC3495771

[btz814-B8] Goldstein E.J. et al (2017) Integrating patient and whole-genome sequencing data to provide insights into the epidemiology of seasonal influenza A(H3N2) viruses. Microb. Genom., 2018, 4. 10.1099/mgen.0.000137 PMC5857367 29310750

[btz814-B9] Holley G., Peterlongo P. (2012) Blastgraph: intensive approximate patternmatching in sequence graphs and De Bruijn graphs In: *Proceedings of the Prague Stringology Conference 2012, Prague, Czech Republic*, pp. 53–63. Available from: http://www.stringology.org/event/2012/p06.html (7 February 2019, date last accessed).

[btz814-B10] Holmes E.C. et al (2005) Whole-genome analysis of human influenza A virus reveals multiple persistent lineages and reassortment among recent H3N2 viruses. PLoS Biol., 3, e300. 16026181 10.1371/journal.pbio.0030300 PMC1180517

[btz814-B11] Houlihan C.F. et al (2018) Use of whole-genome sequencing in the investigation of a nosocomial influenza virus outbreak. J. Infect. Dis., 218, 1485–1489. 29873767 10.1093/infdis/jiy335 PMC6151078

[btz814-B12] Hunt M. et al (2015) IVA: accurate *de novo* assembly of RNA virus genomes. Bioinformatics, 31, 2374–2376. 25725497 10.1093/bioinformatics/btv120 PMC4495290

[btz814-B13] Imai K. et al (2018) Whole genome sequencing of influenza A and B viruses with the MinION sequencer in the clinical setting: a pilot study. Front. Microbiol., 9, 2748. 30483243 10.3389/fmicb.2018.02748 PMC6243006

[btz814-B14] Iuliano A.D. et al (2018) Estimates of global seasonal influenza-associated respiratory mortality: a modelling study. Lancet, 391, 1285–1300. 29248255 10.1016/S0140-6736(17)33293-2 PMC5935243

[btz814-B15] Jonges M. et al (2014) Emergence of the virulence-associated PB2 E627K substitution in a fatal human case of highly pathogenic avian influenza virus A(H7N7) infection as determined by Illumina ultra-deep sequencing. Virology, 88, 1694–1702. 10.1128/JVI.02044-13 PMC3911586 24257603

[btz814-B16] Kim D. et al (2015) HISAT: a fast spliced aligner with low memory requirements. Nat. Methods, 12, 357–356. 25751142 10.1038/nmeth.3317 PMC4655817

[btz814-B18] Leonard A.S. et al (2016) Deep sequencing of Influenza A virus from a human challenge study reveals a selective bottleneck and only limited intrahost genetic diversification. Virology, 90, 11247–11258. 10.1128/JVI.01657-16 PMC5126380 27707932

[btz814-B19] Li H. (2018) Minimap2: pairwise alignment for nucleotide sequences. Bioinformatics, 34, 3094–3100. 29750242 10.1093/bioinformatics/bty191 PMC6137996

[btz814-B20] Li H., Durbin R. (2009) Fast and accurate short read alignment with Burrows-Wheeler transform. Bioinformatics, 25, 1754–1760. 19451168 10.1093/bioinformatics/btp324 PMC2705234

[btz814-B21] Li H. et al (2009) The sequence alignment/map format and SAMtools. Bioinformatics, 25, 2078–2079. 19505943 10.1093/bioinformatics/btp352 PMC2723002

[btz814-B22] Li W., Godzik A. (2006) Cd-hit: a fast program for clustering and comparing large sets of protein or nucleotide sequences. Bioinformatics, 22, 1658–1659. 16731699 10.1093/bioinformatics/btl158

[btz814-B23] Limasset A. et al (2016) Read mapping on De Bruijn graphs. Bioinformatics, 17, 237. 27306641 10.1186/s12859-016-1103-9 PMC4910249

[btz814-B24] Liu B. et al (2016) deBGA: read alignment with De Bruijn graph-based seed and extension. Bioinformatics, 32, 3224–3232. 27378303 10.1093/bioinformatics/btw371

[btz814-B25] McGinnis J. et al (2016) Next generation sequencing for whole genome analysis and surveillance of influenza A viruses. J. Clin. Virol., 79, 44–50. 27085509 10.1016/j.jcv.2016.03.005

[btz814-B26] Meinel D.M. et al (2018) Whole genome sequencing identifies influenza A H3N2 transmission and offers superior resolution to classical typing methods. Infection, 46, 69–76. 29086356 10.1007/s15010-017-1091-3

[btz814-B27] Ondov B.D. et al (2016) Mash: fast genome and metagenome distance estimation using MinHash. Genome Biol., 17, 132. 27323842 10.1186/s13059-016-0997-x PMC4915045

[btz814-B28] Orton R.J. et al (2015) Distinguishing low frequency mutations from RT-PCR and sequence errors in viral deep sequencing data. BMC Genomics, 16, 299. 25886445 10.1186/s12864-015-1456-x PMC4425905

[btz814-B29] Petrova V.N., Russell C.A. (2018) The evolution of seasonal influenza viruses. Nat. Rev. Microbiol., 16, 47–60. 29081496 10.1038/nrmicro.2017.118

[btz814-B30] Rutvisuttinunt W. et al (2013) Simultaneous and complete genome sequencing of influenza A and B with high coverage by Illumina MiSeq platform. J. Virol. Methods, 193, 394–404. 23856301 10.1016/j.jviromet.2013.07.001

[btz814-B31] Salmela L., Rivals E. (2014) LoRDEC: accurate and efficient long read error correction. Bioinformatics, 30, 3506–3514. 25165095 10.1093/bioinformatics/btu538 PMC4253826

[btz814-B32] Sautto G.A. et al (2018) Towards a universal influenza vaccine: different approaches for one goal. Virol. J., 15, 17. 29370862 10.1186/s12985-017-0918-y PMC5785881

[btz814-B33] Sedlazeck F.J. et al (2013) NextGenMap: fast and accurate read mapping in highly polymorphic genomes. Bioinformatics, 29, 2790–2791. 23975764 10.1093/bioinformatics/btt468

[btz814-B34] Shu Y., McCauley J. (2017) GISAID: global initiative on sharing all influenza data - from vision to reality. Euro Surveill., 22, 30494. 28382917 10.2807/1560-7917.ES.2017.22.13.30494 PMC5388101

[btz814-B35] Simon B. et al (2019) Whole genome sequencing of A (H3N2) influenza viruses reveals variants associated with severity during the 2016–2017 season. Viruses, 11, 108. 10.3390/v11020108 PMC6410005 30695992

[btz814-B36] Tafalla M. et al (2016) A comprehensive review of the epidemiology and disease burden of influenza B in 9 European countries. Hum. Vaccin. Immunother., 12, 993–1002. 26890005 10.1080/21645515.2015.1111494 PMC4962970

[btz814-B37] Tange O. (2011) GNU Parallel - The Command-Line Power Tool.; login: The USENIX Magazine. **36**, 42–47. http://www.gnu.org/s/parallel (7 June 2018, date last accessed).

[btz814-B38] Taubenberger J.K., Kash J.C. (2010) Influenza virus evolution, host adaptation, and pandemic formation. Cell Host Microbe, 7, 440–451. 20542248 10.1016/j.chom.2010.05.009 PMC2892379

[btz814-B39] Wan Y. et al (2015) VirAmp: a galaxy-based viral genome assembly pipeline. GigaScience, 4, 19. 25918639 10.1186/s13742-015-0060-y PMC4410580

[btz814-B40] Wood D.E., Salzberg S.L. (2014) Kraken: ultrafast metagenomic sequence classification using exact alignments. Genome Biol., 15, R46. 24580807 10.1186/gb-2014-15-3-r46 PMC4053813

[btz814-B41] Wu N.C. et al (2014) High-throughput profiling of influenza A virus hemagglutinin gene at single-nucleotide resolution. Sci. Rep., 4, 4942. 24820965 10.1038/srep04942 PMC4018626

[btz814-B42] Wymant C. et al (2018) Easy and accurate reconstruction of whole HIV genomes from short-read sequence data with shiver. Virus Evol., 4, vey007. 29876136 10.1093/ve/vey007 PMC5961307

[btz814-B43] Yu X. et al (2014) Influenza H7N9 and H9N2 viruses: coexistence in poultry linked to human H7N9 infection and genome characteristics. Virology, 88, 3423–3431. 10.1128/JVI.02059-13 PMC3957952 24403589

[btz814-B44] Zhou B. et al (2009) Single-reaction genomic amplification accelerates sequencing and vaccine production for classical and swine origin human influenza A viruses. J. Virol., 83, 10309–10313. 19605485 10.1128/JVI.01109-09 PMC2748056

[btz814-B45] Zhou B. et al (2014) Universal influenza B virus genomic amplification facilitates sequencing, diagnostics, and reverse genetics. J. Clin. Microbiol., 52, 1330–1337. 24501036 10.1128/JCM.03265-13 PMC3993638

