# Supplementary material for: Influenza classification from short reads with VAPOR facilitates robust mapping pipelines and zoonotic strain detection for routine surveillance applications
Source: Bioinformatics. 2019 Nov 6;36(6):1681–8. doi: 10.1093/bioinformatics/btz814 (PMC7703727; doi:10.1093/bioinformatics/btz814)
Supplement: btz814_Supplementary_Data [file btz814_supplementary_data.pdf]

SUPPLEMENTARY INFORMATION: Influenza Classification from Short Reads with VAPOR Facilitates Robust Mapping Pipelines and Zoonotic Strain Detection for Routine Surveillance Applications

Mapping Simulations With and Without VAPOR

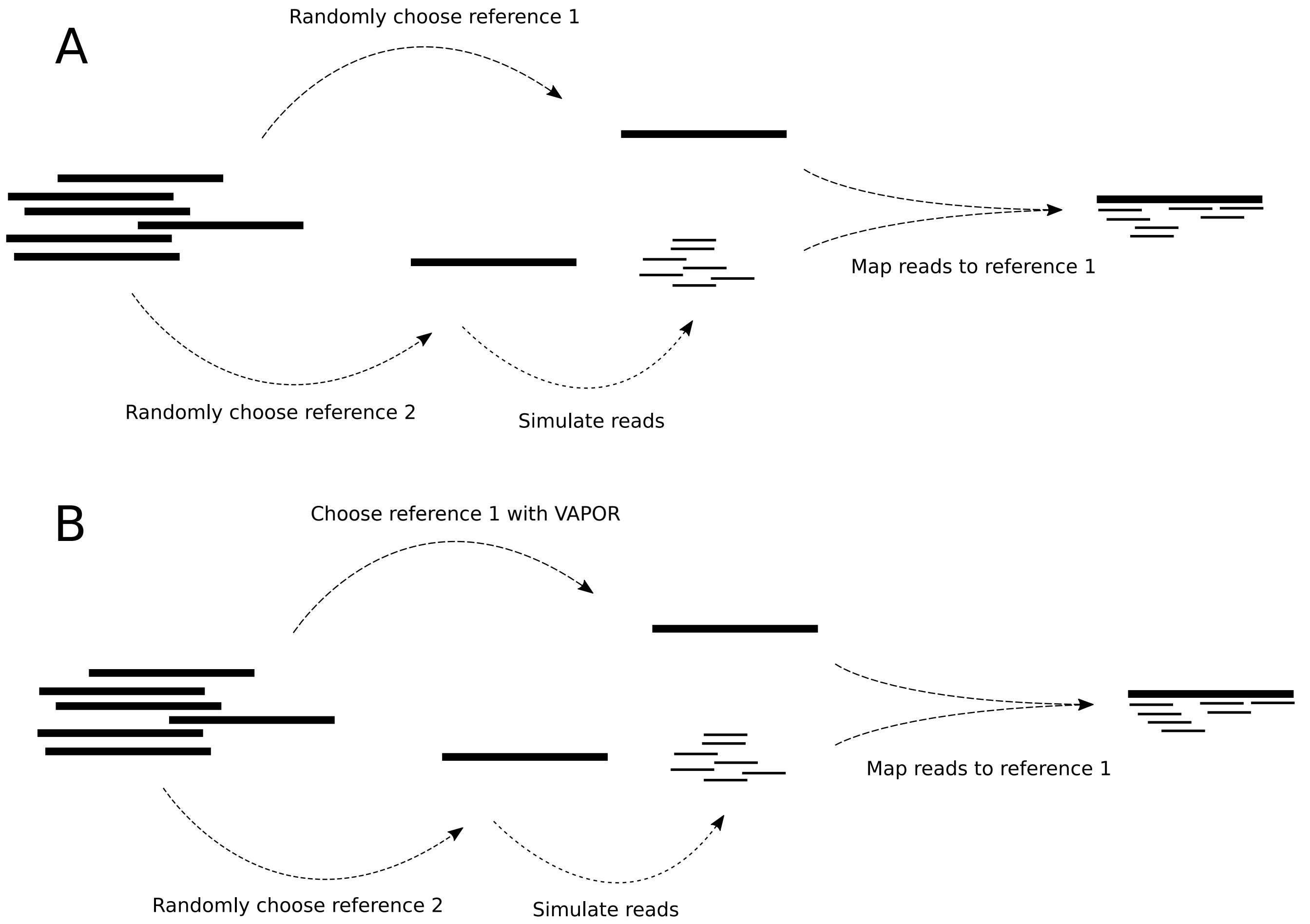

Figure 1: **Random mapping simulations performed with and without reference selection with VAPOR**. In the first case (A), a randomly chosen reference (1) is chosen as a mapping reference, with a second randomly chosen reference (2) used to generate reads. In the second case (B), the mapping reference (1) is selected by VAPOR, instead of being randomly chosen.

Divergence Over Time

In order to assess the accumulation of substitutions over time for purposes of mapping, 21007 H3N2 HA sequences were downloaded from the NCBI Influenza Virus Resource [1], and aligned with MAFFT [2]. As shown in Supplementary Figure 1, the hamming distance of aligned sequences from the earliest sequenced strain, A/Aichi/2/1968, was recorded. Outliers with more than 400 substitutions were excluded.

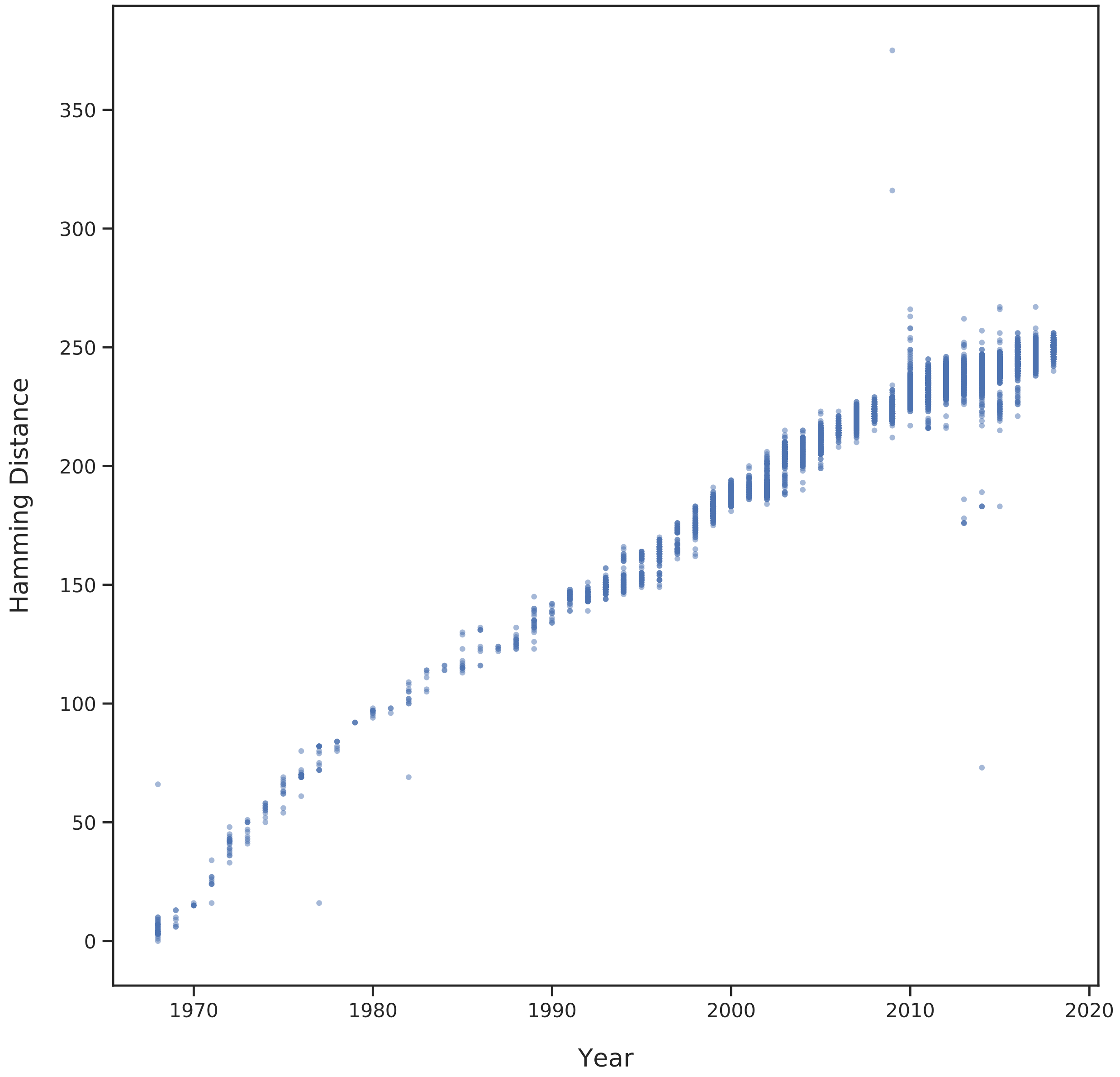

Figure 2: **Hamming distance of aligned H3N2 HA sequences from A/Aichi/2/1968**. On average, sequences accumulate a hamming distance of approximately 5 substitutions per year.
.

Read Error Distribution

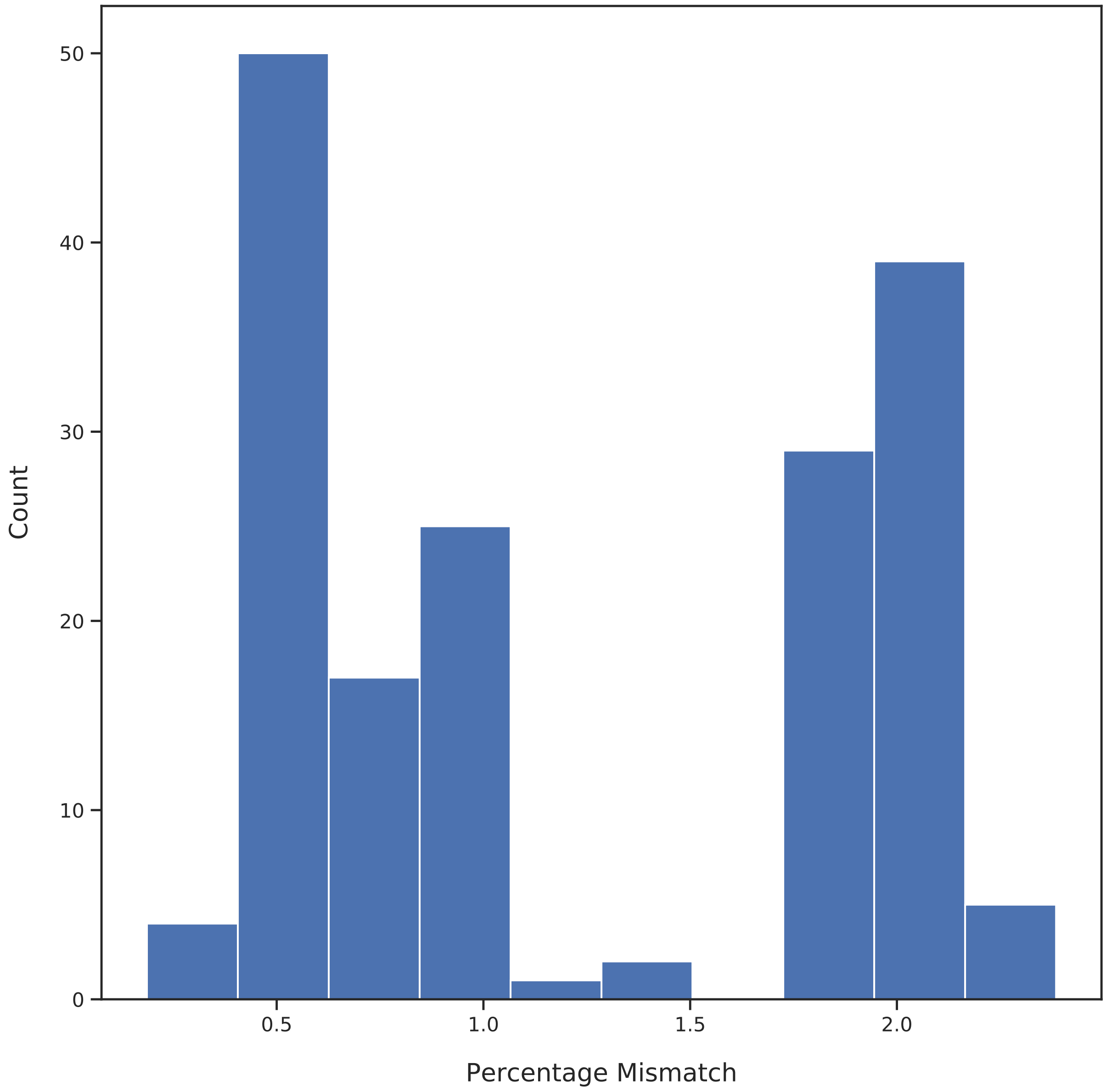

Figure 3: **Histogram giving percentage of mismatches in reads relative to assembled contigs.**
.

Mapping of Divergent H3N2 HA Sequences

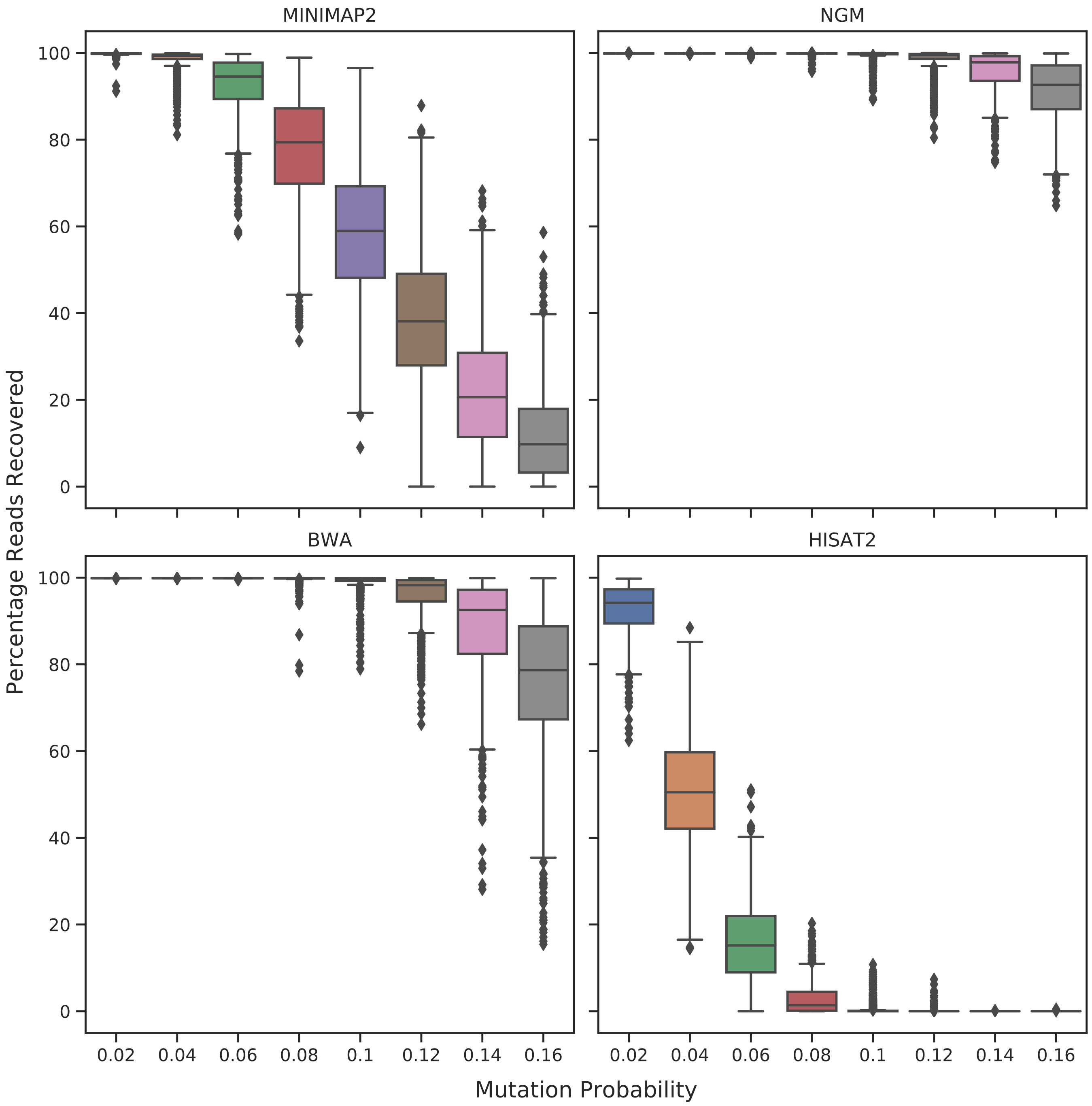

Figure 4: **Box plots showing percentage of simulated Human H3N2 HA reads mapping to Perth/16/2009 for each software, with *in silico* uniform mutation at indicated per-base probability**. Reads were simulated from *in silico* uniformly mutated Perth/16/2009 HA with the indicated per-base probability, approximately corresponding to 2 to 16% divergence. Reads were additionally subjected to 0.05% substitution to account for technical noise, such as from RT-PCR, and biological noise, such as from intrahost variation. Data loss was frequently observed with all tools beyond 10% mutation. Outliers are indicated as diamonds. N=1000 for each category.

Mapping Random Pairs Without Classification

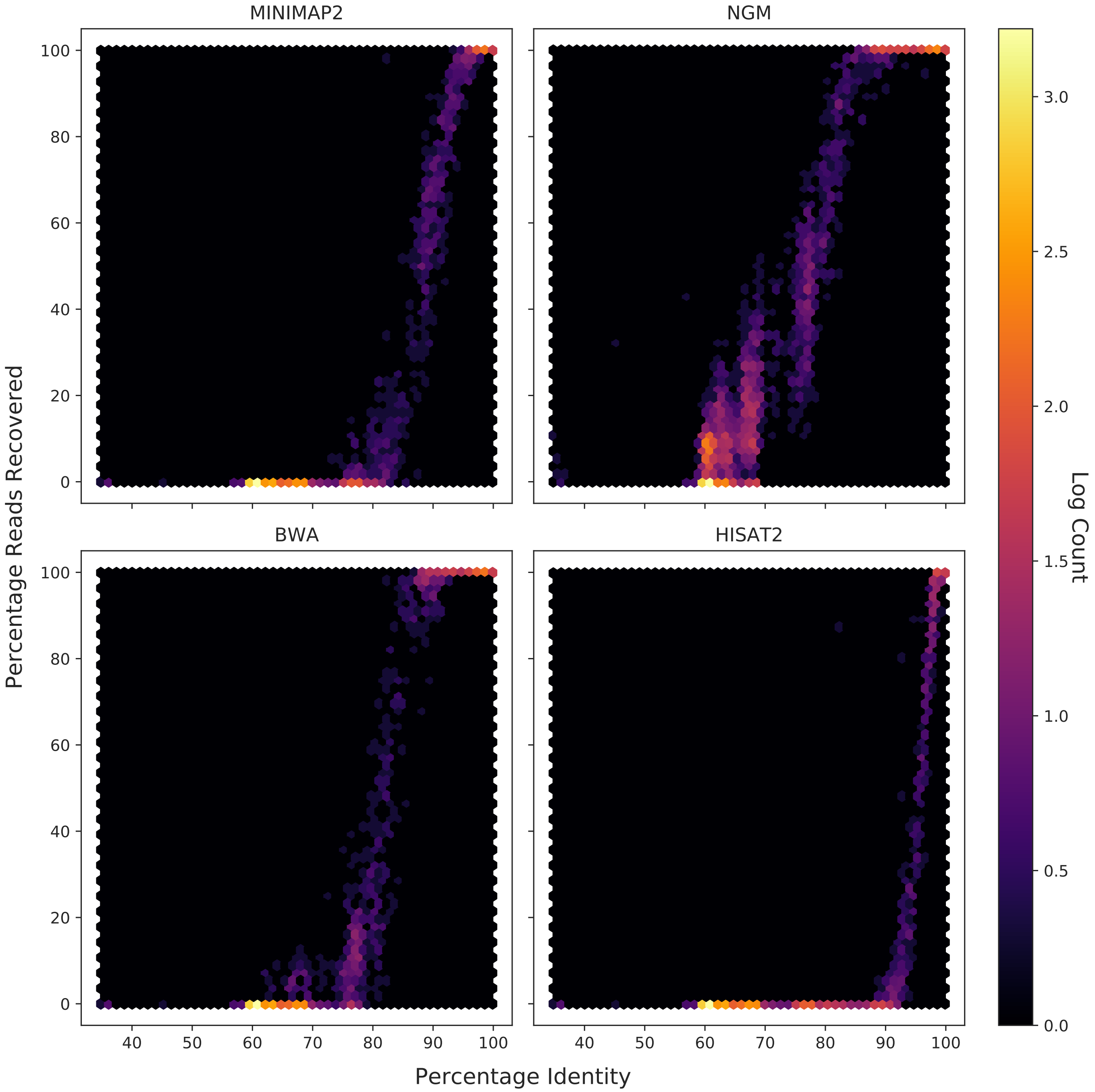

Figure 5: **Read recovery versus percentage identity for randomly chosen influenza A HA sequence pairs**. Influenza A HA sequences were chosen randomly from the NIVR database in pairs, one used as a reference, and one used to simulate reads for mapping. For percentage identities lower than 10%, mapping was unreliable. Furthermore, notably between strains of differing host origin, percentage identity was observed in many cases to be as low as 60%. These trends were observed for all tools, although performance dropped off more gradually for NGM.

Reassortment Detection

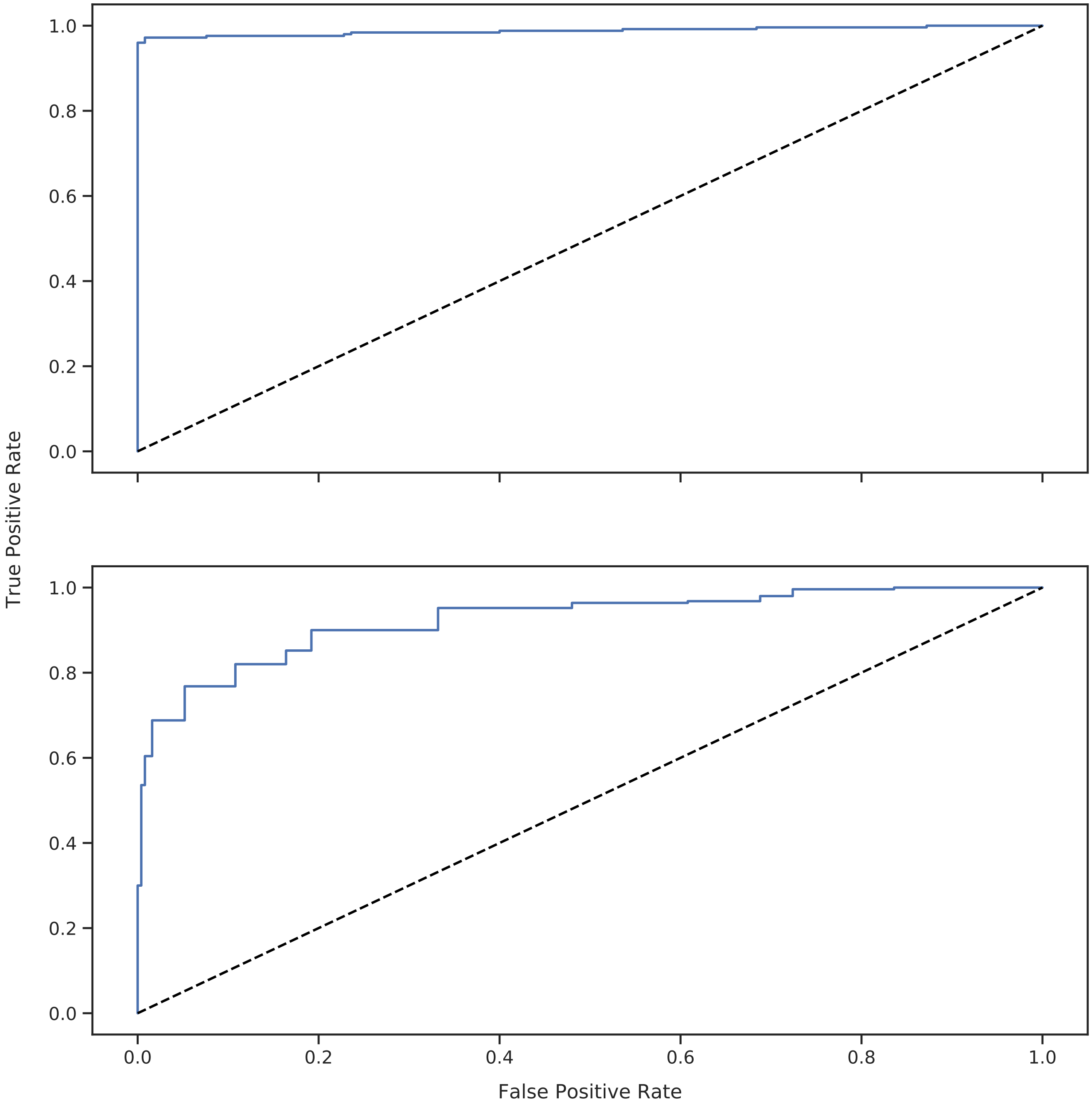

Figure 6: **Receiver operating characteristic (ROC) curve for classification of simulated reassortment events**. Although most zoonotic reassortments were detected at all parameter values (top), intra-H3N2 lineage (bottom) reassortments were more difficult to detect. The curve was generated by varying v, the minimum PID between VAPOR classifications of individual segment strains on the basis of HA. Due to the noise present in VAPOR classifications, as well as the close sequence similarity of H3N2 sequences, all parameter values with high TPR corresponded to a large FPR.

Run-time Benchmarks

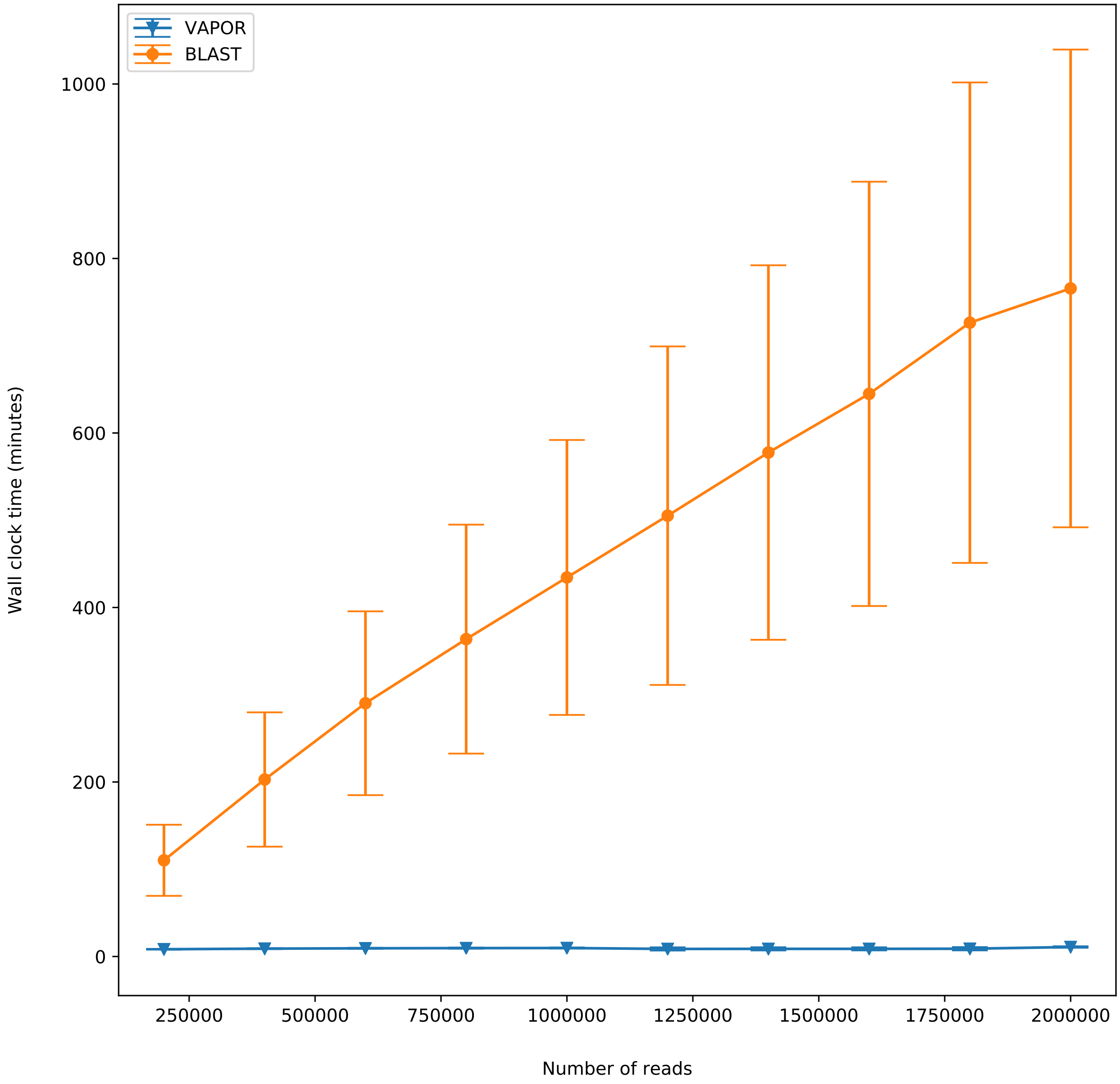

Figure 7: **Run-time for BLAST and VAPOR for classification of influenza reads.** A reference database of 47,073 influenza A HA gene sequences was used. Error bars give the sample standard deviation.

References

[1] Bao, Y., Bolotov, P., Dernovoy, D., Kiryutin, B., Zaslavsky, L., Tatusova, T., Ostell, J., Lipman, D.: The influenza virus resource at the National Center for Biotechnology Information. Journal of Virology **82**, 596–601 (2008)

[2] Katoh, K., Standley, D.: MAFFT multiple sequence alignment software version 7: Improvements in performance and usability. Molecular Biology and Evolution **30**, 772–780 (2013)
